# Supplementary material for: A host E3 ubiquitin ligase regulates Salmonella virulence by targeting an SPI‐2 effector involved in SIF biogenesis
Source: mLife. 2023 Jun 13;2(2):141–58. doi: 10.1002/mlf2.12063 (PMC10989757; doi:10.1002/mlf2.12063)
Supplement: Supplementary file 1 — Supporting information. [file MLF2-2-141-s001.pdf]

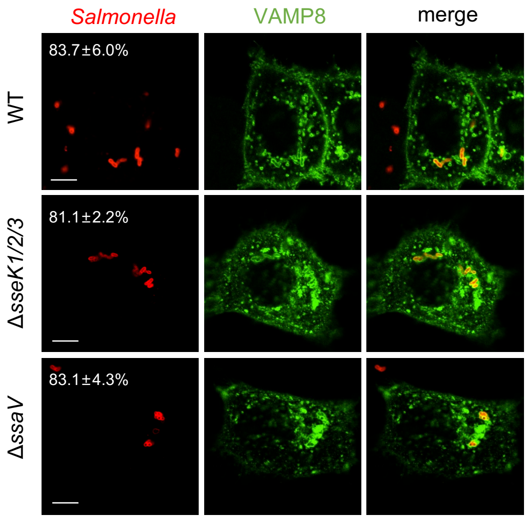

Figure S1. The Effects of SseKs on the frequency of VAMP-8 coated *Salmonella*. EGFP-VAMP8 expressed HeLa cells were infected with the indicated *Salmonella* strains for 2 hr. Shown are fluorescence detection of VAMP8 (green) and *Salmonella* (red). Statistics of cells showing the frequency of VAMP-8 coated *Salmonella* are listed in the upper left corner. At least 100 cells were counted for each experiment, and the statistical data shown are from three independent determinations. Scale bar, 10 µm.

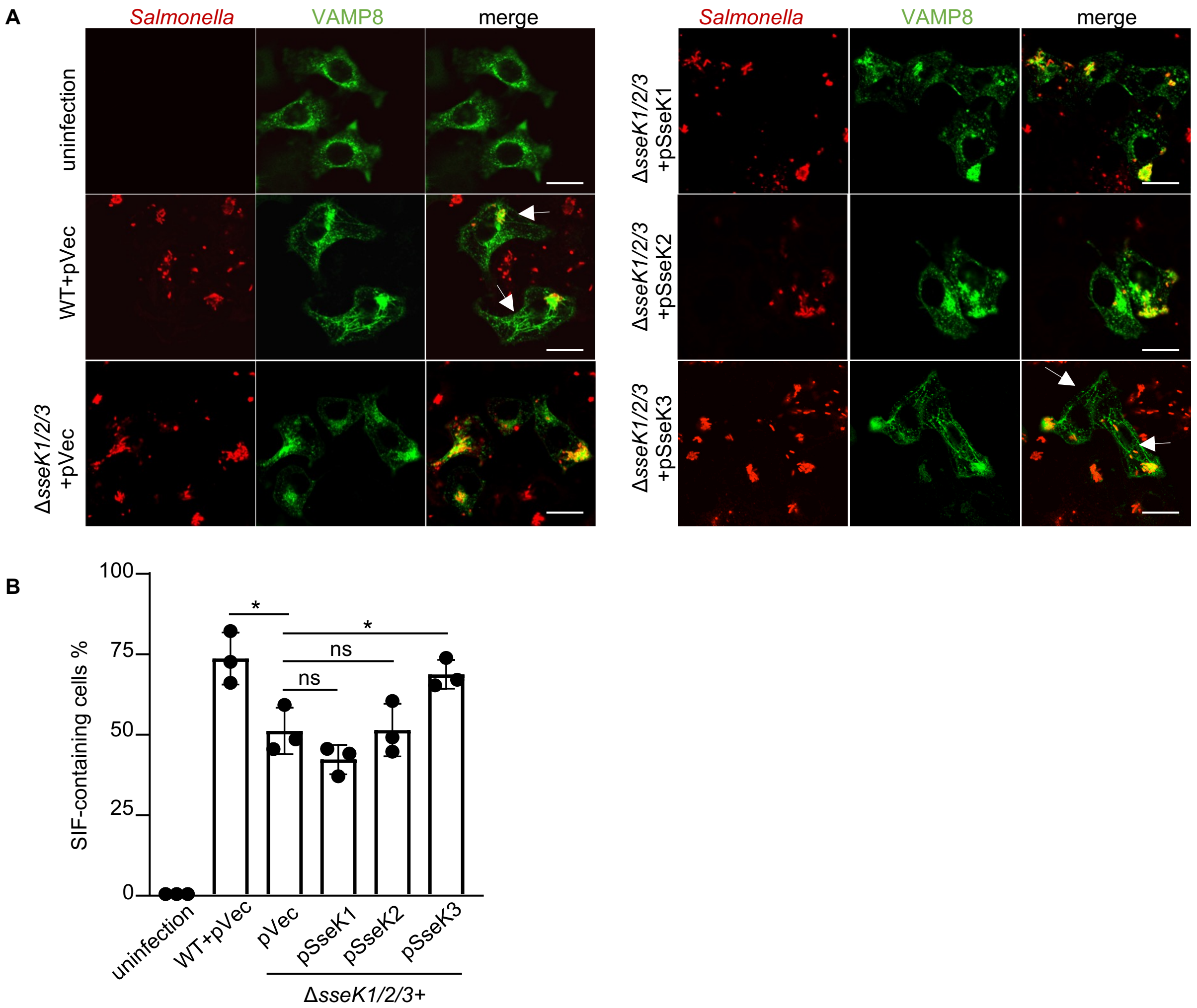

Figure S2. SseK3, but not SseK1 or SseK2, could rescue SIF-formation defects of the Δ*sseK1/2/3* mutant during *S.* Typhimurium infection. HeLa cells expressing EGFP-VAMP8 were infected with the indicated *S.* Typhimurium strains for 10 h and analyzed for SIFs. **(A)** Representative images show VAMP8 (green) distribution and *S.* Typhimurium (red). The white arrow indicates the SIF structure. Scale bar, 25 µm. **(B)** The rates of VAMP8-positive tubules for each sample are indicated. At least 50 cells were counted for samples from experiments done in triplicate. **P*<0.05

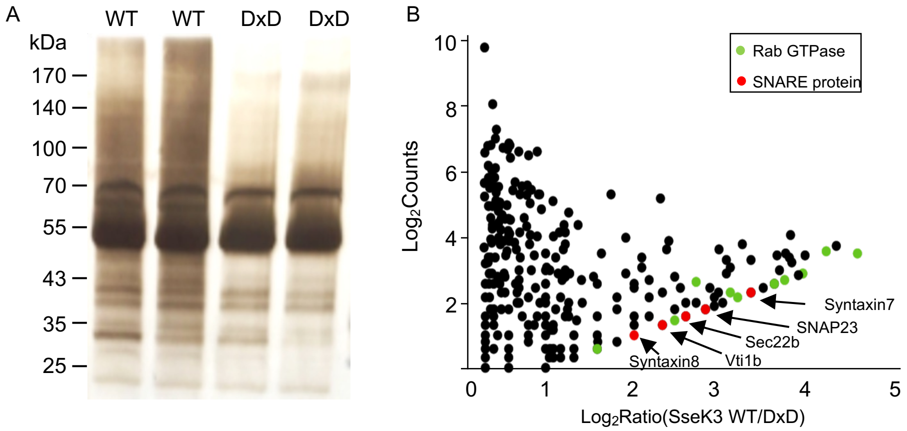

Figure S3. Over-expression of SseK3 GlcNAcylates host SNARE proteins. (A) Detection of enriched Arg-GlcNAcylated proteins by silver staining. Lysates of 293T cells transfected to express GFP-SseK3 (WT) or GFP-SseK3 (DxD) were subjected to immunoprecipitation with Arg-GlcNAc-specific antibodies, precipitates separated by SDS-PAGE were detected by silver staining from two independent experiments.(B) Scatter plots of protein ratios as a function of their relative abundance. Proteins immunoprecipitated with an anti-Arg-GlcNAc antibody were subjected to LC-MS/MS analysis. The ratio was calculated as spectral counts in SseK3 (WT)-transfected samples divided by those in SseK3 (DxD)-transfected samples. Large ratios indicate preferential detection and modification in 293T cells transfected to express SseK3. Red dots correspond to SNARE proteins, and green dots correspond to Rab GTPase proteins. This data corresponds to Fig.1C.

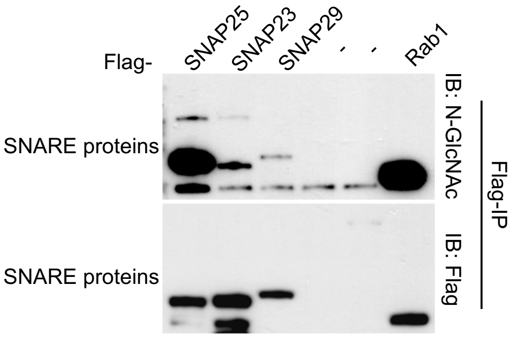

Figure S4. **Modification of SNAP proteins by SseK3 during *S.* Typhimurium infection**. 293T cells were transfected with a plasmid expressing the Flag-SNAP23, Flag-SNAP25, Flag-SNAP29 or Flag-Rab1 individually, and then infected with *S.* Typhimurium Δ*sseK1/2/3* complemented with pET28a-SseK3. After infection, cells were lysed, and proteins were immunoprecipitated with anti-Flag beads, followed by standard immunoblotting analysis with the indicated antibodies.

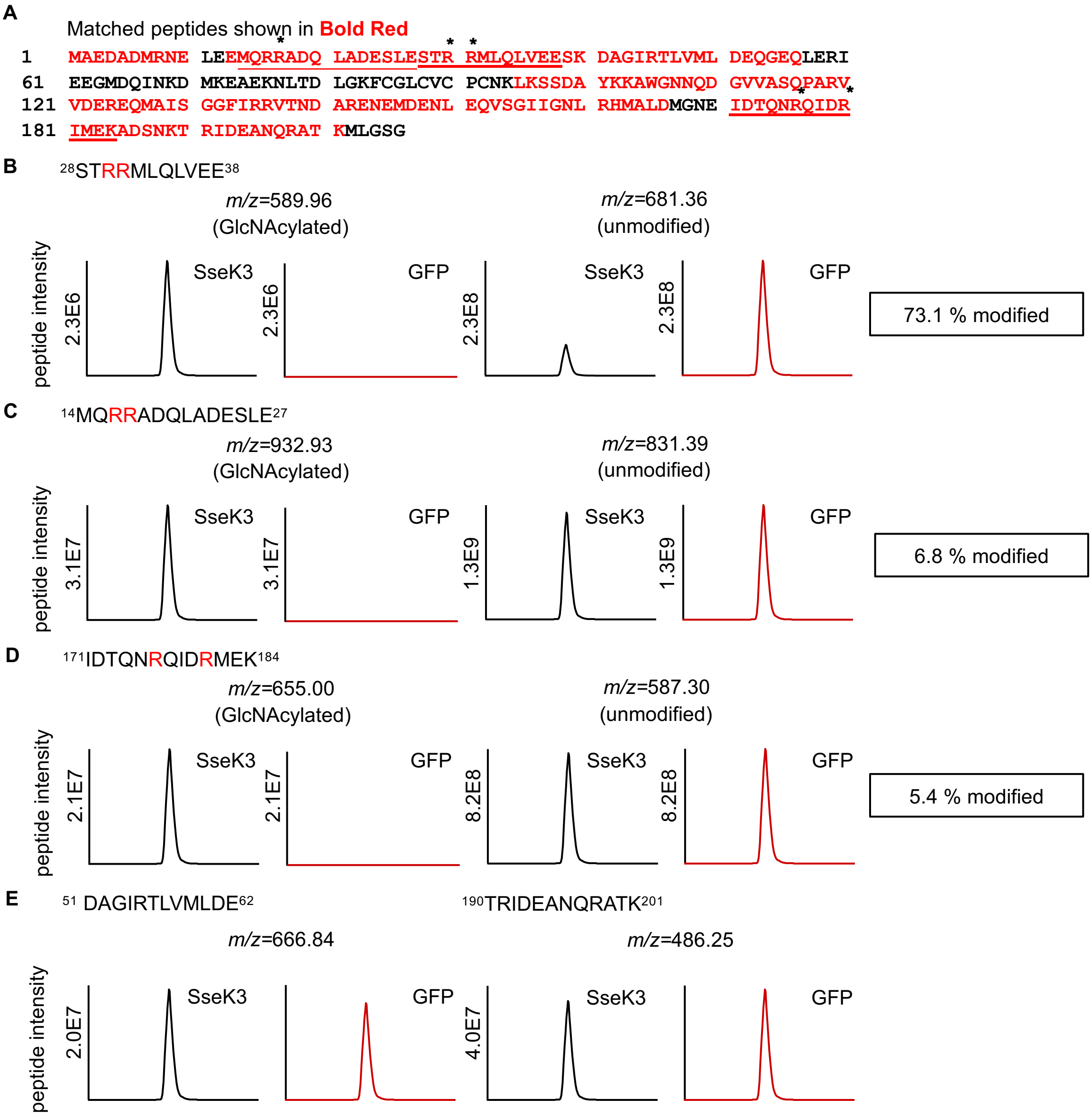

Figure S5. MS detection of SNAP25 peptide intensities. (A)Flag-SNAP25 was isolated from 293T cells co-transfected with either wild-type GFP-SseK3 or the empty plasmid GFP. Immunoprecipitated SNAP25 was then digested with GluC and LysC, and then analyzed by LC-MS/MS. Detected SNAP25 sequence shown in red in LC-MS experiments. The GlcNAcyalted peptide sequences are underlined, and the modification sites are indicated by asterisks. The peptides ^{28}STRRMLQLVEE^{38} (B), ^{14}MQRRADQLADESLE^{27} (C), and ^{171}IDTQNRQIDRMEK^{184} (D) covalently modified with GlcNAc molecule are shown, and the detected arginines are labeled in red. Two control peptides are displayed in (E). Extracted ion chromatograms of the doubly protonated peptide are shown with peak intensities indicating the relative amounts of either the modified or unmodified peptides. These data correspond to Fig 2A.

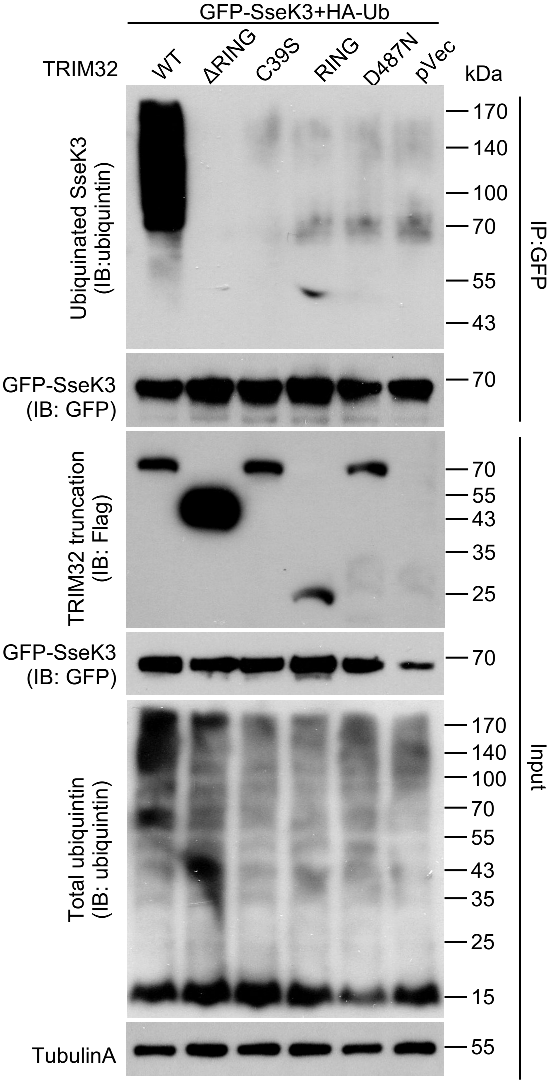

Figure S6. Overexpression of wild-type but not the mutant TRIM32 promotes ubiquitination of SseK3. 293T cells transfected to express GFP-SseK3 and TRIM32 or its mutants in the presence of HA-ubiquitin. 18 h after transfection, co-immunoprecipitation was performed with anti-GFP antibodies, followed by standard immunoblotting analysis with the indicated antibodies.

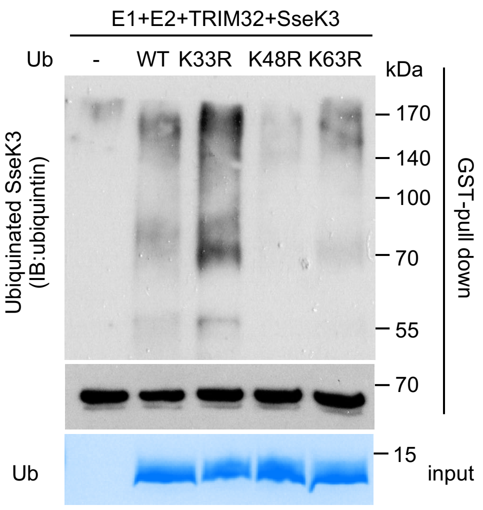

Figure S7. TRIM32 catalyzes SseK ubiquitinatination *in vitro*. Recombinant His-TRIM32, GST-SseK3, ubiquitin, E1 (huBE1), and E2 (UBCH5c) were added as indicated for ubiquitination assays. After GST pull-down, ubiquitin-conjugated proteins were detected by immunoblot with a ubiquitin-specific antibody. The input levels of Ub proteins were detected by Coomassie brilliant blue staining. Data shown are a representative of three independent experiments with similar results.
