# Supplementary material for: A host E3 ubiquitin ligase regulates Salmonella virulence by targeting an SPI‐2 effector involved in SIF biogenesis
Source: mLife. 2023 Jun 13;2(2):141–58. doi: 10.1002/mlf2.12063 (PMC10989757; doi:10.1002/mlf2.12063)
Supplement: Supplementary file 2 — Supporting information. [file MLF2-2-141-s002.docx]

**Supplementary Information for**

A host E3 ubiquitin ligase regulates Salmonella virulence by targeting an SPI-2 effector involved in SIF biogenesis

Kun Meng^1#^, Jin Yang^1#^, Juan Xue^1^, Jun Lv^1^, Ping Zhu^1^, Liuliu Shi^4^, Shan Li^1,2,3^*

^1^Institute of Infection and Immunity, Taihe Hospital, Hubei University of Medicine, Shiyan, Hubei, China;

^2^ State Key Laboratory of Agricultural Microbiology, College of Life Science and Technology, Huazhong Agricultural University, Wuhan, Hubei, China;

^3^College of Biomedicine and Health, Huazhong Agricultural University, Wuhan, Hubei, China; ^4^School of Basic Medical Science, Hubei University of Medicine,Shiyan,China.

* Corresponding author: Shan Li

**Email:** lishan@mail.hzau.edu.cn

**This PDF file includes:**

Figures S1 to S7

Table S1 to S3

SI References

**SupplementaryTables**

**Table S1. Bacterial strains used in this study**

| Strain or plasmid | Relevant characteristics | References |
| --- | --- | --- |
| BL21(DE3) | used for protein expression/purification | Novagen |
| DH5α | used for cloning | Novagen |
| SL1344 | wild type *S*.Typhimurium, Strep^r^ | ATCC |
| SL1344 Δ*ssaV* | *ssaV* gene deleted in SL1344, Strep^r^ | [1] |
| SL1344 Δ*sseK1/2/3* | *sseK1*,*sseK2* and *sseK3* gene deleted in SL1344, Strep^r^ | [1] |
| SL1344 Δ*sseK1/2/3+*pET28a | Δ*sseK1/2/3* containing pET28a, Strep^r^, Km^r^ | [1] |
| SL1344 Δ*sseK1/2/3+*pSseK1 | Δ*sseK1/2/3* containing pET28a-SseK1, Strep^r^, Km^r^ | [1] |
| SL1344 Δ*sseK1/2/3+*pSseK2 | Δ*sseK1/2/3* containing pET28a-SseK2, Strep^r^, Km^r^ | [1] |
| SL1344 Δ*sseK1/2/3+*pSseK3 | Δ*sseK1/2/3* containing pET28a-SseK3, Strep^r^, Km^r^ | [1] |
| SL1344 Δ*sseK1/2/3+*pSseK3 DxD | Δ*sseK1/2/3* containing pET28a-SseK3 DxD, Strep^r^, Km^r^ | [1] |

**Table S2. Plasmids used in this study**

| Plasmids | Relevant characteristics | References |
| --- | --- | --- |
| pCS2-GFP-SseK3 | pCS2-GFP carrying SseK3 coding region, Amp^r^ | [1] |
| pCS2-GFP-SseK3 DxD | pCS2-GFP carrying SseK3 D226A/D228A mutation, Amp^r^ | [1] |
| pCS2-RFP-SseK3 | pCS2-RFP carrying SseK3 coding region, Amp^r^ | [1] |
| pCS2-Flag-SNAP23 | pCS2-Flag carrying SNAP23 coding region, Amp^r^ | This study |
| pCS2-Flag-SNAP25 | pCS2-Flag carrying SNAP25 coding region, Amp^r^ | This study |
| pCS2-GFP-SNAP25 | pCS2-GFP carrying SNAP25 coding region, Amp^r^ | This study |
| pCS2-Flag-SNAP25 NTD | pCS2-Flag carrying SNAP25 (1-90 aa) region, Amp^r^ | This study |
| pCS2-HA-SNAP23 | pCS2-HA carrying SNAP23 coding region, Amp^r^ | This study |
| pCS2-HA-SNAP25 | pCS2-HA carrying SNAP25 coding region, Amp^r^ | This study |
| pCS2-Flag-VAMP8 | pCS2-Flag carrying VAMP8 coding region, Amp^r^ | This study |
| pCS2-HA-VAMP8 | pCS2-HA carrying VAMP8 coding region, Amp^r^ | This study |
| pcDNA4-GFP-VAMP8 | pcDNA4 carrying GFP-VAMP8 coding region, Amp^r^ | This study |
| pCS2-Flag-Vti1b | pCS2-Flag carrying Vti1b coding region, Amp^r^ | This study |
| pCS2-Flag-Sec22b | pCS2-Flag carrying Sec22b coding region, Amp^r^ | This study |
| pCS2-Flag-Snapin | pCS2-Flag carrying Snapin coding region, Amp^r^ | This study |
| pCS2-Flag-Syntaxin 7 | pCS2-Flag carrying Syntaxin7 coding region, Amp^r^ | This study |
| pCS2-Flag- Syntaxin 8 | pCS2-Flag carrying Syntaxin8 coding region, Amp^r^ | This study |
| pCS2-Flag- Rab1 | pCS2-Flag carrying Rab1 coding region, Amp^r^ | [1] |
| pCS2-Flag-TRIM32 WT | pCS2-Flag carrying TRIM32 (1-653aa) region, Amp^r^ | This study |
| pCS2-Flag-TRIM32 RING | pCS2-Flag carrying TRIM32 (1-96 aa) region, Amp^r^ | This study |
| pCS2-Flag-TRIM32 RING+B-box | pCS2-Flag carrying TRIM32 (1-135 aa) region, Amp^r^ | This study |
| pCS2-Flag-TRIM32 NHL | pCS2-Flag carrying TRIM32 (255-653 aa) region, Amp^r^ | This study |
| pCS2-Flag-TRIM32 ΔRING | pCS2-Flag carrying TRIM32 (97-653 aa) region, Amp^r^ | This study |
| pCS2-Flag-TRIM32 Δ(RING+B-box) | pCS2-Flag carrying TRIM32 (136-653 aa) region, Amp^r^ | This study |
| pCS2-Flag-TRIM32 ΔNHL | pCS2-Flag carrying TRIM32 (1-254 aa) region, Amp^r^ | This study |
| pCS2-Flag-TRIM32 C39S | pCS2-Flag carrying TRIM32 C39S mutation, Amp^r^ | This study |
| pCS2-Flag-TRIM32 R394H | pCS2-Flag carrying TRIM32 R394H mutation, Amp^r^ | This study |
| pCS2-Flag-TRIM32 D487N | pCS2-Flag carrying TRIM32 D487N mutation, Amp^r^ | This study |
| pHKO14-Cas9 | SpCas9 expression plasmid, blasticidin resistance | Gang Cao’s lab |
| pHKO-GFP-sgRNA | sgRNA cloning backbone for expression of sgRNA in mammalian cells, puromycin resistance | Gang Cao’s lab |
| pRK5-HA-Ub WT | pRK5-HA carrying wild type ubiquitin | Addgene |
| pRK5-HA-Ub K27R | pRK5-HA carrying ubiquitin K27R mutation | This study |
| pRK5-HA-Ub K29R | pRK5-HA carrying ubiquitin K29R mutation | This study |
| pRK5-HA-Ub K33R | pRK5-HA carrying ubiquitin K33R mutation | This study |
| pRK5-HA-Ub K48R | pRK5-HA carrying ubiquitin K48R mutation | This study |
| pRK5-HA-Ub K63R | pRK5-HA carrying ubiquitin K63R mutation | This study |
| pRK5-HA-Ub K48 only | all Lys residues are mutated to Arg except K48 | [2] |
| pRK5-HA-Ub K63R only | all Lys residues are mutated to Arg except K63 | [2] |

**Table S3. Antibodies and reagents used in this study**

| **REAGENT or RESOURCE** | SOURCE | IDENTIFIER |
| --- | --- | --- |
| Antibodies | | |
| Mouse monoclonal anti-tubulinA | Sigma-Aldrich | Cat#T5168 |
| Rabbit polyclonal anti-Arg-GlcNAc | Abcam | Cat#ab195033 |
| Mouse monoclonal anti-Flag | Sigma-Aldrich | Cat#F7425 |
| Mouse monoclonal anti-EGFP | Santa Cruz Biotechnology | Cat#sc8334 |
| Mouse monoclonal anti-HA | Biolegend | Cat#901501 |
| Rabbit polyclonal anti-Salmonella | Abcam | Cat#ab35156 |
| Mouse monoclonal anti-Ubiquitin P4D1 | Santa Cruz Biotechnology | Cat#sc8017 |
| Mouse monoclonal anti-ATP1B1 | Santa Cruz Biotechnology | Cat#sc21713 |
| Mouse monoclonal anti-Tom20 | Santa Cruz Biotechnology | Cat#sc17764 |
| Mouse monoclonal anti-GM130 | BD Biosciences | Cat#5239872 |
| Goat Anti-Rabbit IgG H&L (Alexa Fluor^®^ 488) | Thermo Fisher Scientific | A32731 |
| Goat Anti-Mouse IgG H&L (Alexa Fluor^®^ 488) | Thermo Fisher Scientific | A32723 |
| Goat Anti-Rabbit IgG H&L (Alexa Fluor® 564) | Thermo Fisher Scientific | A-11035 |
| Goat Anti-Mouse IgG H&L (Alexa Fluor® 564) | Thermo Fisher Scientific | A-11030 |
| Goat Anti-Rabbit IgG H&L (Alexa Fluor^®^ 647) | Thermo Fisher Scientific | A32733 |
| Goat Anti-Mouse IgG H&L (Alexa Fluor^®^ 647) | Thermo Fisher Scientific | A32728 |
| Chemicals, peptides, and recombinant proteins | | |
| DAPI | Sigma-Aldrich | Cat#D9542 |
| MG-132 | Sigma-Aldrich | Cat#C2211 |
| Chloramphenicol | Sigma-Aldrich | Cat#R4408 |
| Isopropyl β-D-Thiogalactoside (IPTG) | Sigma-Aldrich | Cat#I6758 |
| Cycloheximide | Sigma-Aldrich | Cat#C7698 |
| Ampicillin | Sigma-Aldrich | Cat#A9393 |
| Gentamicin | Sigma-Aldrich | Cat#G1397 |
| Kanamycin | Sigma-Aldrich | Cat#K1377 |
| Dulbecco’s Modified Eagle’s Medium, High Glucose | Gibico | Cat#C11885500BT |
| PBS pH 7.4 basic (1×) | Gibco | Cat#C10010500BT |
| Fetal Bovine Serum | Biological Industries | Cat#04-001-1ACS |
| 0.05% Trypsin-EDTA | Thermo Fisher Scientific | Cat#25300054 |
| Complete Protease Inhibitor | Roche | Cat#11836170001 |
| DAPI | Thermo Fisher Scientific | Cat#D1306 |
| jetPRIME^®^ | Polyplus transfection | Cat#114-15 |
| VigoFect | Vigorous biotechnology | Cat#T001 |
| Trypsin Protease, MS Grade | Thermo Fisher Scientific | Cat#90057 |
| Lys-C Endoproteinase, MS Grade | Thermo Fisher Scientific | Cat#90051 |
| Glu-C Endoproteinase | Thermo Fisher Scientific | Cat#90054 |
| EZview^TM^ Red ^®^ANTI-FLAG M2 Affinity Gel | Sigma-Aldrich | Cat#F2426 |
| Anti-GFP Affinity Beads 4FF | SMART LIFESCIENCES | Cat#SA070001 |

**SI References**

1. Meng K, et al. (2020) Arginine GlcNAcylation of Rab small GTPases by the pathogen Salmonella Typhimurium. Communications Biology 3(1):287.

2. Yang Q*, et al.* (2017) TRIM32-TAX1BP1-dependent selective autophagic degradation of TRIF negatively regulates TLR3/4-mediated innate immune responses. *PLoS pathogens* 13(9):e1006600.
